# Breast Self-Examination and breast cancer awareness in women in developing countries: a survey of women in Buea, Cameroon

**DOI:** 10.1186/1756-0500-5-627

**Published:** 2012-11-09

**Authors:** Mary Atanga Bi Suh, Julius Atashili, Eunice Asoh Fuh, Vivian Ayamba Eta

**Affiliations:** 1 Department of Nursing, Faculty of Health Sciences, University of Buea, Buea, Cameroon; 2 Department of Public Health and Hygiene, Faculty of Health Sciences, University of Buea, Buea, Cameroon

**Keywords:** Breast cancer, Breast Self-Exam, Knowledge, Practices, Cameroon

## Abstract

**Background:**

Breast cancer is one of the leading causes of cancer morbidity and mortality worldwide. In Cameroon, breast cancer causes as many as 10.7 deaths per 100,000 women making it the second cause of cancer mortality. Better documenting women’s knowledge and practices on breast cancer and breast self-exam (BSE) would be useful in the design of interventions aimed at preventing breast cancer. This study sought to 1. describe Cameroonian women’s knowledge of breast self-examination (BSE); 2. assess their impression on the practice of BSE and 3. describe their perceptions on the causes, risk factors and prevention of breast cancer.

**Methods:**

A cross-sectional survey was conducted in a volunteer sample of 120 consenting women in Buea, Cameroon. Data were collected using a structured questionnaire self-administered by study participants.

**Results:**

The sample was fairly educated with close to three quarters (70.83%) having completed high school. Nearly three quarters (74.17%) of participants had previously heard about BSE, however as many as 40% had never done a BSE. Although 95% of participants believed that breast cancer could be prevented, only 36.67% recognized breast examination as a prevention method. A substantial 13.33% thought that breast cancer could be prevented with a vaccine while 45% thought that dieting or exercising would prevent breast cancer. Similarly, 70% of participants thought that breast cancer could be treated, with 35.83% thinking that it could be treated medically while 34.17% thought it could be treated traditionally or spiritually.

**Conclusions:**

The practice of BSE while perceived as being important is not frequent in these women in Buea, Cameroon. Health education campaigns are imperative to elucidate the public on the causes, risk factors and prevention of breast cancer. Further studies need to explore what interventions could be best used to improve the uptake and practice of BSE.

## Background

Breast cancer is the second cause of cancer worldwide and the fifth cause of cancer mortality [1]. It is estimated that the prevalence of breast cancer in women aged 15 and over in sub-Saharan Africa was 23.5 per 100,000 women in 2008 [2]. During the same period an estimated 35,427 women died from breast cancer - a crude mortality rate of 12.8 per 100.000 women [3]. In Cameroon the crude annual incidence of breast cancer is estimated at 19.3 per 100,000 women with a mortality of 10.7 per 100,000 women [3].

Prevention remains the cornerstone of the fight against breast cancer worldwide. Although some prevention methods have been proposed, many remain inaccessible to women in developing countries who, ironically, given the limited diagnostic and curative facilities available to them, need prevention the most. Breast self-examination (BSE), although not having been shown to be effective in reducing mortality [4], is still recommended as a general approach to increasing breast health awareness and thus potentially allow for early detection of any anomalies [5]. Furthermore, BSE continues to be recommended by health care practitioners because it is free, painless and easy to practice. Despite this potential, there is very little data indicating the uptake and practice of BSE in Cameroon. Little is known concerning women’s knowledge about breast cancer, their knowledge on BSE and their practice of BSE.

Better documenting women’s knowledge on breast cancer and BSE as well as their practice of BSE would be useful in the design of interventions aimed at preventing breast cancer through increased awareness and or improved screening. We thus conducted this study to 1. describe Cameroonian women’s knowledge of breast self-examination (BSE); 2. assess their impression on the practice of BSE and 3. describe their perceptions on the causes, risk factors and prevention of breast cancer.

## Methods

### Study design and setting

We conducted a descriptive cross-sectional study in Buea, Cameroon. Buea is the administrative headquarter of the South West Region of the Republic of Cameroon. It has an estimated population of 200,000 people. The town is comprised of a multi-ethnic population, with almost all the 254 ethnic groups found in Cameroon represented in the area, attracted by the fertile volcanic soil good for agriculture. The diverse composition of this population was suitable for this study.

The population is primarily served by the Buea Regional Hospital, an intermediate level health facility which has a radiology department as well as a radiologist but no mammography equipment or any system for systematically screening women for breast cancer. There is no oncologist and women diagnosed with breast cancer need to be referred to Douala or Yaounde which are respectively approximately 35 and 160 miles from Buea.

### Study population, sampling and participants

The target population comprised of women in Buea. To be eligible women had to be aged 20 years or more, consenting and be willing to fill a self-administered questionnaire. Participants were identified by convenient and consecutive sampling of eligible women met either at their job sites or the streets of Buea. Recruitment was pursued until the required sample size of 120 was reached.

### Data collection, variables and measurements

A standardized questionnaire was used to collect data. This questionnaire had three sections: demographic characteristics, knowledge and impressions about BSE, and a section on knowledge about breast cancer. The questionnaire was self-administered: consenting participants were given printed copies of the questionnaire and allowed time to fill their responses at their will and convenience, and in a private, confidential setting. Participants then returned these questionnaires anonymously to the researcher.

The questionnaires were self-administered so as to limit bias and allow for forthright responses from participants. Given the relatively semi-urban and literate nature of the study population, no participant was excluded because of the inability to read or write. The sample size of 120 was chosen by convenience.

### Data management and statistical analysis

Data from the questionnaire were entered into a spreadsheet and analysed using Microsoft Excel. Based on the purely descriptive nature of the study we described continuous variables such as age using means and standard deviations, and categorical variables such as the frequency of yes, no responses using their frequencies and the percentages.

### Ethical considerations

This study was conducted as part of a requirement of an academic degree and received required ethical and administrative approval from the Faculty of Health Sciences, University of Buea, Cameroon.

This manuscript was written following STROBE guidelines for the reporting of observational studies [6].

## Results

### Study participants

The characteristics of the 120 women who participated in this study are summarised in Table 1. A plurality (41.67%) of the women were aged between 20-30 years and approximately half (51.66%) were single. The sample was fairly educated with close to three quarters (70.83%) having completed high school. Slightly less than a third (28.33%) of the sample was made of students while another third (30.00%) were self-described business women. A vast majority of women were Christians (76.66%) and self-described Bantus (69.17%).

**Table 1:** Characteristics of 120 women who responded to survey on Breast Self-examination and Breast Cancer in Buea, Cameroon

**Characteristic**	**Level**	**Frequency**	**%**
Age (years)			
	20-30	50	41.67
	31-40	38	31.67
	41-50	22	18.33
	50 and above	10	8.33
Marital Status			
	Married	50	41.67
	Single	62	51.66
	Divorced	3	2.5
	Widow	5	4.17
Occupation			
	Student	34	28.33
	Business woman	36	30.00
	Farmer	8	6.67
	Housewife	16	13.33
	Civil servant	26	21.67
Education level			
	Primary school	21	17.5
	Secondary school	14	11.67
	High school	51	42.5
	University degree	34	28.33
Religion			
	Christians	92	76.66
	Muslims	5	4.17
	Others	23	19.17
Ethnicity			
	Bantus	83	69.17
	Fulbe	11	9.17
	Semi- Bantus	26	21.67

### Participants’ knowledge, practice and perceptions on breast self-examination

Nearly three quarters (74.17%) of participants had previously heard about BSE (Table 2). Approximately 6 in every 10 women (59.17%) claimed to know how to perform BSE with 35% reporting performing BSE monthly and 12.5% performing it 6-monthly. As many as 40% of participants had never done a BSE.

**Table 2:** Knowledge, practice and perceived importance of breast self-examination in 120 women in Buea, Cameroon

**Knowledge/Practice**	**Response**	**Frequency**	**%**
Ever heard about BSE			
	Yes	89	74.17
	No	31	25.83
Know how to perform BSE			
	Yes	71	59.17
	No	49	40.83
Frequency of BSE			
	Monthly	42	35.00
	Every 6 months	15	12.5
	Yearly	15	12.5
	Never	48	40.00
Overall Awareness on BSE*			
	Not aware	31	25.83
	Partially aware	47	39.17
	Substantially aware	42	35.00
Impression on importance of BSE			
	Important	114	95
	Not important	6	5

* Not aware: never heard of BSE, had absolutely no idea on how to perform it and never practiced it; partially aware: heard of BSE, had slight idea on how to perform it but did not practice it often; substantially aware: heard of BSE, knew how to perform it and practiced it often.

Overall 25.6% of respondents were not aware of BSE (they had never heard of BSE, had absolutely no idea on how to perform a BSE and never practiced it) while 39.17% were partially aware (had heard of BSE, had a slight idea on how to perform it but did not practice it often) and 35% were substantially aware of BSE (had heard of BSE, knew how to perform it and practiced it often).

Despite a substantial proportion of women not being aware of BSE nearly all of them (95%) recognised the importance of BSE for their health.

### Participants’ knowledge and perceptions on breast cancer

All the participants had knowledge of the existence of breast cancer. Participants’ perceptions about the causes, risk factors, prevention and treatment of breast cancer are summarised in Table 3.

**Table 3:** Knowledge of and perceptions about breast cancer in 120 women in Buea, Cameroon

**Knowledge/Perception**	**Frequency**	**%**
**Knowledge of breast cancer’s existence**	120	100.00
**Perceived causes of Breast Cancer**		
Exposure to a wide range of cancer causing agents	64	53.33
A family history of breast cancer	29	24.17
Exposure to x-rays before 30 years of age	22	18.33
Overweight after menopause	12	10.00
Prolonged and early use of oral contraceptives	16	13.33
Oestrogen replacement therapy	14	11.67
Witchcraft	12	10.00
**Perceived Risk factors for Breast Cancer**		
Excessive alcohol consumption	24	20.00
Excessive cigarette smoking	40	33.33
Inactivity and sedentary lifestyle	23	19.17
Prolonged use of dark hair dyes	15	12.50
High animal fat diet	18	15.00
Consumption of milk products from cows injected with genetically engineered hormones	26	21.67
**Perception that Breast cancer can be prevented**	114	95.00
**Perceived methods of preventing Breast cancer**		
Dieting	30	25.00
Exercise	24	20.00
Vaccination	16	13.33
Breast examination	44	36.67
**Perception that Breast cancer can be treated**	84	70.00
**Perceived methods of treating breast cancer**		
Medically	43	35.83
Traditionally	11	9.17
Spiritually	30	25.00

The most frequent perceived cause of breast cancer was “exposure to a wide range of cancer causing agents” (53.33%). Breast cancer was attributed to witchcraft by 10% of the participants. The risk factors most frequently indexed by participants were “excessive cigarette smoking” (33.33%), consumption of genetically modified products (21.67%), excessive alcohol consumption (20.00%) and an inactive sedentary lifestyle (19.17%). Overall 51.77% of participants were partially aware of the causes of breast cancer (recognised at least two accepted causes of breast cancer) while 48.33% were substantially aware (recognised five or more commonly accepted causes of breast cancer). On the other hand 5% of respondents were not aware of risk factors of breast cancer (did not know any breast cancer risk factor), while 50% were partially aware (recognized at least two accepted breast cancer risk factors) and 45% were substantially aware (recognized five or more accepted breast cancer risk factors).

Although 95% of participants believed that breast cancer could be prevented, only 36.67% recognized breast examination as a breast cancer prevention method. A substantial 13.33% thought that breast cancer could be prevented with a vaccine while 45% thought that dieting or exercising would prevent breast cancer.

Similarly, 70% of participants thought that breast cancer could be treated, with 35.83% thinking that it could be treated medically while 34.17% thought it could be treated traditionally or spiritually.

## Discussion

In this study we showed that although a vast majority of women in a semi-urban area of Cameroon knew about breast cancer, many had misperceptions on its risk factors, prevention and treatment. Furthermore, despite three-quarters reporting having heard about BSEs less than a quarter reported practicing it.

To the best of our knowledge this is the first report of community participants’ perceptions on BSE and breast cancer in Cameroonian women. Our findings were similar to those of these other studies of African women in showing considerable awareness about the existence of breast cancer, but poor knowledge on the risk factors and causes of breast cancer, as well as infrequent and inconsistent practice of BSE in various groups of women in Nigeria, Angola, Senegal and South Africa [7-17]. Specifically, in Nigeria, a study of female secondary students in Abuja indicated that while a high proportion knew about BSE being used to prevent breast cancer very few (10.1%) practiced it [15]. Similarly, close to three quarters of female undergraduate students in Nigeria had heard about BSE, although only about one in five had ever practiced it [18]. Still in Nigeria, a study of rural women revealed poor knowledge similarly perceptions about a mystical origin of breast cancer such as it being due an “attack from the enemy” [19]. Further, a study of women in a semi-urban area of Nigeria (Osogbo), found that despite close to three quarters being aware of breast cancer lees than half could identify associated symptoms or risk factors [9]. In Angola, a study of 595 university students indicated that a majority of them where not knowledgeable about breast cancer [17]. In Senegal a survey of 300 women indicated only 42.7% having knowledge about BSE and only 29% practicing it [12]. The situation appeared worse in women of African descent in South Africa in whom a survey indicated that close to one-fifth of women had not heard of breast cancer and half were not aware of BSE [8].

Our findings are confined to a sample of semi-urban and relatively educated women, thus limiting their generalizability to the whole of Cameroon. The use of self-administered questionnaires may have accounted for the higher level of education. Knowledge about breast cancer and BSE may be much lower in rural areas. The study findings may also be limited by the fact that these were based on self-reports – women were not asked nor assessed on their ability to correctly perform a BSE, thus the estimate of knowledge of how to perform a BSE may be an overestimate. The convenient sample size of 120 may also imply imprecision in the estimates derived.

## Conclusion

Overall our findings indicate that the practice of BSE while perceived as being important is not frequently practiced in these women in Buea, Cameroon. Considering the substantial role that can be played by BSE in such settings with limited prevention methods (mammography is unavailable) combined with the high burden of breast cancer, these findings suggest an urgent need for interventions to implement and re-enforce existing cancer awareness and cancer screening programs. Health education campaigns will be needed to elucidate the public on the causes, risk factors and prevention of breast cancer. These education and information sessions would also need to dispel myths such as that witchcraft playing a role in the genesis of breast cancer while also inculcating the substantial role of genetics and unknown factors. Further studies need to explore what interventions could be best used to improve the uptake and practice of BSE and other methods for early breast cancer detection.

## Competing interest

The authors declare no conflict of interest.

## Authors’ contributions

Study conception and design: MABS, EAF; Study implementation: MABS, EAF; Analysis and review: EAF, JA, VEA; Manuscript writing and revisions: JA, MABS, VEA. All authors read and approved the final manuscript.
